# An assessment of WRF-urban schemes in simulating local meteorology for heat stress analysis in a tropical sub-Saharan African city, Lagos, Nigeria

**DOI:** 10.1007/s00484-024-02627-3

**Published:** 2024-02-16

**Authors:** Oluwafemi Benjamin Obe, Tobi Eniolu Morakinyo, Gerald Mills

**Affiliations:** 1 University College Dublin, Dublin, Ireland; 2 Institute of Future Cities, Chinese University of Hong Kong, Hong Kong, Hong Kong SAR

**Keywords:** Lagos, Heat stress, Local climate zone, Humidex, WRF-urban, Sub-Saharan Africa

## Abstract

Megacities, such as Lagos, Nigeria, face significant challenges due to rapid urbanization and climate change, resulting in a higher intensity of the urban heat island effect, coupled with high population density, making the city fall under the category of moderate to high heat stress/risk. Despite this, very few studies have analyzed the urban impact on heat stress over the coastal city, albeit with poor resolution data. In this study, we assessed the performance of an integrated high-resolution WRF-urban scheme driven by the readily available urban canopy information of the local climate zone (LCZ) to simulate local meteorological data for analyzing the spatiotemporal pattern of heat stress over the megacity. Our results show that the WRF-BEP scheme outperformed the other evaluated urban schemes, reducing the normalized root mean squared error by 25%. Furthermore, using humidex, we found a generally high incidence of intense discomfort in highly urbanized areas and noted the significant influence of urban morphology on the pattern of heat stress, particularly at night due to the combined effect of urban warming and higher relative humidity. The most socioeconomically disadvantaged urban areas, LCZ7, were most affected, with “hot” heat stress conditions observed over 90% of the time. However, during the afternoon, we found reduced heat stress in the core urban areas which might be due to the shading effect and/or cold air advection. Our findings would be relevant in the development of the urgently needed climate/heat adaptation plans for the city and other sub-Saharan African cities.

**Supplementary Information:**

The online version contains supplementary material available at 10.1007/s00484-024-02627-3.

## Introduction

Urbanization is rapidly reshaping the global landscape, primarily fueled by economic growth in urban areas (Moriconi-Ebrard et al. 2016; Saghir & Santoro 2018). African megacities in particular, are expected to become epicenters of urban expansion before the end of the century (Antwi-Afari et al. 2021; Castells-Quintana & Wenban-Smith 2020). Sub-Saharan Africa, in particular, is witnessing substantial population growth as a result of rapid development of it urban centers further exacerbating the urbanization process (Hoornweg & Pope 2014; Li et al. 2021). Urbanization in major cities such as Lagos Nigeria have brought significant changes to the natural landscape thereby transforming once moist and permeable surfaces into dry and impermeable ones (Wang & Maduako 2018). This transformation has given rise to the urban heat island (UHI) effect, resulting in localized temperature increase within the urban cores, which, in turn, impact microclimates, intensify heatwaves, and contribute to heat stress-related effects.

Global awareness of the impact of urbanization on local and regional temperature increase has grown, with studies showing that urbanization is a significant driver of UHI effects in cities across the globe (e.g., Du et al. 2016; Kardinal Jusuf et al. 2007; Zhou et al. 2014). Furthermore, projections indicate that increasing global temperatures will lead to more frequent and severe heatwaves in urban areas (Dosio 2017), further exacerbated by the UHI effects, making equatorial regions, like sub-Saharan Africa, particularly vulnerable to heat stress. The link between UHI and heat stress and their direct association with premature deaths and health complications during extreme heat events underscores the urgent need for comprehensive assessments of these hazards and potential mitigation strategies. Even if global warming is limited to 1.5 °C, as per the Paris Agreement, it is projected that cities like Lagos will still experience heat stress, putting millions of residents at risk by 2050 (Fotso-Nguemo et al. 2023). Unfortunately, many cities in this region are ill-prepared to cope with the projected heat stress hazards. This necessitates the assessment of heat stress patterns resulting from urbanization in these regions. While heat stress in urban areas are influenced by meteorological factors such as changes in temperature, humidity, and wind patterns (Juzbašić et al. 2022; Wilhelm Kirch and RB 2005), they also vary locally depending on the nature of urban forms and functions and therefore make different urban areas exposed and vulnerable differently. High-resolution assessments are therefore crucial to understanding these patterns. While acknowledging the global consensus on these issues, critical gaps in data and insights persist, particularly in sub-Saharan Africa context. Comprehensive assessments of heat stress patterns resulting from urbanization in these areas are essential to identify areas requiring mitigation and to safeguard urban populations. However, data gaps pose a significant challenge in these areas.

Observational datasets are commonly employed to study UHI and heat stress patterns (e.g., Basara et al. 2008; Ioannou et al. 2021; McAllister et al. 2022). However, their application has limitations in regions with sparse weather networks (Acuto & Susan 2016; Roth 2007; Good et al. 2017). These limitations arise due to the spatial inefficiency of such datasets for analyzing heat stress patterns. For instance, Ojeh et al. (2016) used air temperature sensors at only two locations in Lagos, revealing a substantial UHI intensity of up to 7 °C. However, this approach, while highlighting the overall UHI magnitude in the city, lacked insights into spatial and intra-urban variabilities in the complex urban environment of Lagos. Another recent study on heat stress in Lagos (Obiefuna et al. 2021) estimated the Universal Thermal Climate Index (UTCI) using Landsat imagery. The results identified locations with strong outdoor heat stress (UTCI > 32 °C), such as Agege, Ifako-Ijaiye, and Ikeja. However, due to data limitations, this study averaged relative humidity and wind speed over the entire day, failing to consider the diurnal variations of heat stress linked to the intricate interplay of urban warming, relative humidity, and wind patterns. Furthermore, this study used Land Surface Temperature (LST) as a proxy for air temperature, despite the significant variability between LST and actual air temperature, particularly across seasons (Vancutsem et al. 2010). Besides these studies, there is a noticeable absence of comprehensive heat stress investigations in Lagos, primarily attributable to the lack of high-resolution weather data.

Recent advances in state-of-the-art atmospheric models have contributed to filling this crucial gap in global UHI and heat stress research. Urban climate researchers have harnessed numerical models’ increased capabilities to capture processes within the urban canopy (Kondo et al. 2005; Mills 1997), even at building scales. Nonetheless, applying such models and obtaining realistic urban canopy data for sub-Saharan African cities remain a significant challenge. In Lagos, for example, a study by Bassett et al. (2020) focused on UHI modeling, utilizing the generic urban canopy parameterization of Jackson et al. (2010). While showcasing the spatiotemporal extent of UHI over three decades, this study relied on a generic urban canopy parameterization. Given the unique local characteristics of the urban landscape in sub-Saharan Africa (Van de Walle et al. 2021; Obe et al. 2023), this approach may not accurately capture the significant impact of landscape heterogeneity in the region.

Building on these insights, our objective is to improve the representation of the 3D urban surface in urban canopy models to simulate local meteorological variables more accurately for comprehensive heat stress studies. For this, the urban canopy parameterization of the local climate zone (Stewart & Oke 2012; Stewart et al. 2014) is used. The LCZ scheme (further described in “Mapping the local climate zone of Lagos Nigeria”) is a classification system that attempts to define urban areas into 10 built-up types of relatively uniform urban morphological configurations and 7 natural land cover types (Stewart and Oke 2012; Stewart et al. 2014). Most importantly, the scheme provides basic information about the urban canopy which could be used in modeling.

Moreover, over sub-Sahara Africa, given the possibility of adopting this scheme, it is still unclear which of the urban canopy models integrated with the Weather Research and Forecasting (WRF) models will perform better in simulating basic meteorological variables (air temperature, relative humidity, and wind patterns) for heat stress analysis. As this is the first paper to use WRF model in estimating heat stress within Lagos metropolis, we robustly assess the performance of different urban canopy models coupled to WRF (described in “Materials and methods”) in simulating basic meteorological variables needed for heat stress evaluation.

The objective of this study is therefore to first assess the performance of multilayer UCMs integrated with WRF (driven with the LCZ scheme) and secondly to examine the spatiotemporal pattern of heat stress in Lagos metropolis. This study is presented as a case study for regional study of the application of WRF-urban scheme in data-sparse region of sub-Sahara Africa for heat stress assessment using humidex based on previous studies that showed the strong association between physiological parameters and humidex, particularly in dense and highly humid coastal cities (e.g., Gosling et al. 2014; Ho et al. 2016; Simpson et al. 2023).

## Materials and methods

### Study area

Lagos, Nigeria, is taken as a typical city to illustrate a hot-humid tropical city, located in West Africa on the Gulf of Guinea and within a tropical savanna climate with pronounced wet and dry seasons according to the Köppen climate classification. The dry season lasts for about 5 months, from December to April, with an average daily high temperature above 32 °C. The hottest month of the year in Lagos is March, with an average maximum temperature of 32 °C (Adejuwon & Odekunle 2006; Odekunle et al. 2005; Odekunle 2010).

**Fig. 1:**
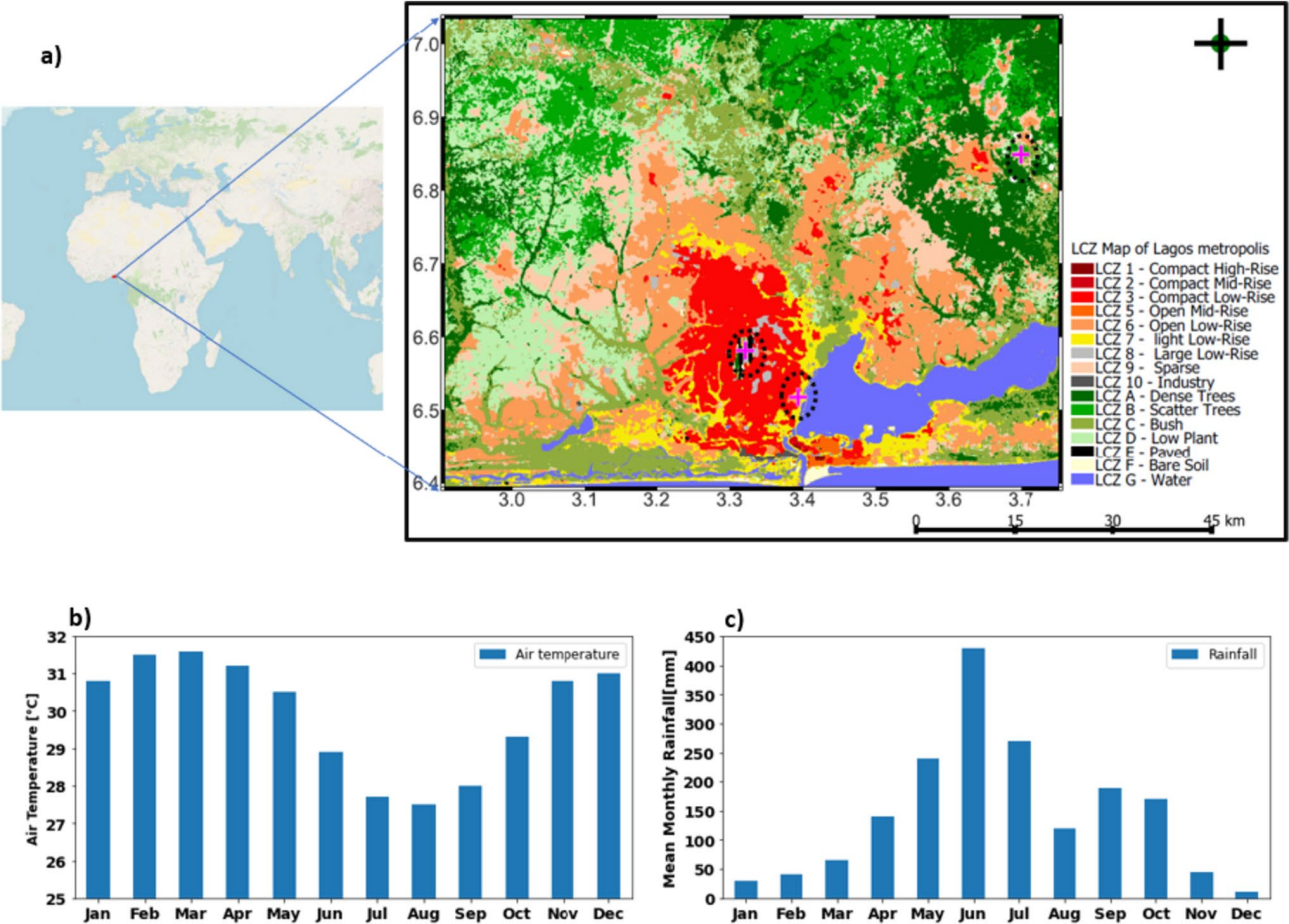
**a** The local climate zone map of Lagos metropolitan area which encompasses Lagos city and a few towns in Ogun state. Note: background image from Open Street Map, the pink cross signs indicates the locations of the three weather stations whose data are used for evaluation. **b** Climatology of monthly maximum temperature for Lagos (1960–2010: adapted from Sojobi et al. 2015)

Lagos is a coastal city whose weather is largely influenced by the humid southwesterlies airmass from the Atlantic making the weather highly humid for most months of the year. As shown in Fig. 1b, Lagos rainfall pattern exhibits a bimodal distribution, with the primary peak occurring in June and a secondary milder peak around October (Odekunle 2010). The city’s sky conditions exhibit variation throughout the year. Clear skies are most prevalent from November to February, transitioning to scattered to broken clouds between March and April. Meanwhile, the wet season is generally characterized by overcast conditions (Adewusi et al. 2015).

Geographically, Lagos urban area exhibits remarkable landscape heterogeneity (Obe et al. 2023) with varying urban forms ranging from towering high-rise buildings on the islands to densely packed shacks made from corrugated metal sheets in informal areas on the mainland. Additionally, Lagos land use consists of over 40% of water bodies and wetlands, which largely limit its expansion. Thus, it is a highly urbanized and highly dense city with a rapidly growing population of over 21 million people within a landmass of 3577 km^2^ making it one of the fastest-growing cities and densest in Africa and in the world. Rapid expansion in Lagos has led to increased land use changes, such as deforestation and land reclamation (Ajibade 2017), and has expanded beyond its administrative boundaries (Badmos et al. 2020; Sawyer 2014) especially in the northwards adjoining towns and suburbs in the neighboring Ogun state in what is now referred to as Lagos metropolis.

### Methodological framework

**Fig. 2:**
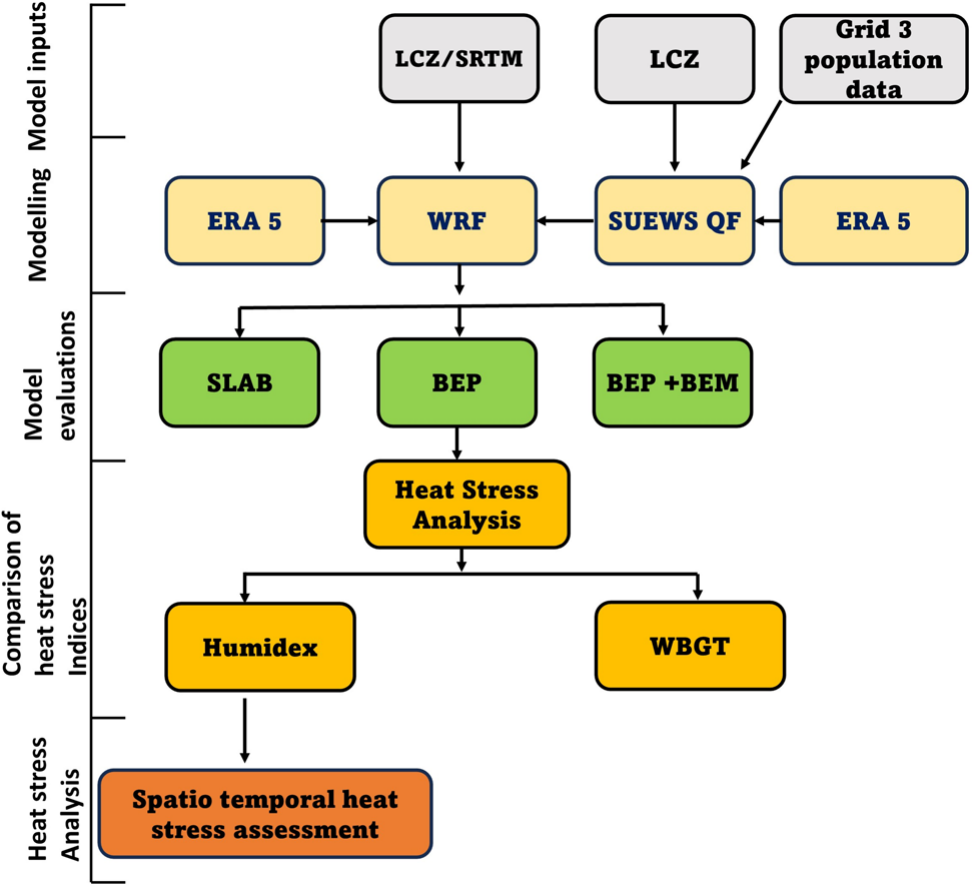
Summarized methodological framework

The methodological workflow of this study is depicted in Fig. 2, starting with the development of LCZ maps which classify the urban landscape into different climate zones with unique urban canopy parameters. This combined with SUEWS-derived anthropogenic heat flux based on GRID3 population data was fed into the Weather Research Forecasting (WRF) model to simulate the meteorological conditions of a pre-monsoon period. Details of the model configuration, model inputs, and simulation period is elaborated in subsequent subsections.

#### Mapping the local climate zone of Lagos, Nigeria

We first developed an LCZ map of Lagos for the year 2020 using the LCZ generator (Demuzere et al. 2021a, b). To generate the map, training areas (TAs) were identified using Google Earth Pro, resulting in a total of 349 TAs. Notably, we utilized urban slum shapefiles from Badmos et al. (2020) to delineate informal areas as LCZ 7. These areas are characterized by high-density shanties constructed predominantly from corrugated metal sheets. Access to these areas is mostly via small murram alleys and typical unpaved roads (Van de Walle et al. 2021; Badmos et al. 2019, 2020; Simon et al. 2013). In general, LCZ 7 had the largest portion of the total TAs (18%), followed by LCZ 6 (15%) and LCZ 3 (15%), LCZ 9 accounted for 9% of the TAs, while LCZs 15, 16, and 1 each represented only 1% of the total TAs. The distribution of TAs across different LCZs corresponded to the area covered by each LCZ. Next, the TAs were supplied to the LCZ generator. In addition to the TAs, the LCZ generator uses state-of-the-art Earth Observation satellite imageries, providing robust input features at a high resolution of 100 m (Demuzere et al. 2021a, b). The resulting map underwent Gaussian filtering following Demuzere et al. (2020). Fig. S1 illustrates the number of TAs per LCZ and their corresponding overall accuracies (OA). The overall accuracy of the LCZ map was found to be 0.63.

The resulting LCZ map (Obe 2022) shown in Fig. 1a illustrates 17 distinct classes, comprising 10 urban and 7 natural categories. In the urban area of Lagos, LCZ 6 dominates with 42%, followed by LCZ 9 at 25%, and LCZ 3 at 17%. LCZ 7 closely follows with 13%, while other urbanized LCZs each constitute less than 2%.

Moreover, a comparative analysis with the global LCZ map (Demuzere et al. 2022) shown in supplementary information (Fig. S2 and Table S1) reveals variations. LCZ 9 holds the majority of the urbanized areas covering 48%, followed by LCZ 6 at 33%, and LCZ 3 at 16%. A detailed comparison table is provided in the Supplementary Information. A notable dissimilarity arises concerning the underrepresentation of informal settlements (LCZ 7) in the global LCZ map. Our generated LCZ map with locally derived TAs significantly improves the representation of these areas. It notably captures a substantial portion depicting slums and informal settlements (LCZ 7), aligning more closely with the actual landscape of the region. This improvement underscores the efficacy of our approach in providing a more accurate depiction of urban characteristics, particularly informal settlements, compared to the global LCZ map.

#### WRF model description

As earlier mentioned, this study uses the Weather Research and Forecasting (WRFv4.4) model (Skamarock et al. 2008) to analyze heat stress over Lagos. However, the study first seeks to assess the performance of multilayer urban canopy models (UCMs) driven with the LCZ scheme in simulating local meteorological data for heat stress analysis over the study area. The UCMs include Simple Layer Urban Canopy (SLAB) model embedded within the Noah land surface model (de la Paz et al. 2016; Liu et al. 2006), BEP (Martilli et al. 2002), and BEP + BEM (Salamanca & Martilli 2010). The SLAB model is a straightforward UCM that treats the urban geometry as a homogeneous flat surface with a large roughness length and small albedo and estimates fluxes of heat and momentum regardless of the specific urban morphological and radiative characteristics (Kusaka and Kimura 2001). The BEP is a 3D structure model that distributes the exchanges of heat, moisture, momentum, and turbulent kinetic energy within the urban structure at different vertical levels. Similar to BEP, the BEP + BEM is a highly sophisticated 3D multilayer scheme that estimates anthropogenic heat generated by air conditioning systems and heat exchange between the interior of a building and the atmosphere.

**Fig. 3:**
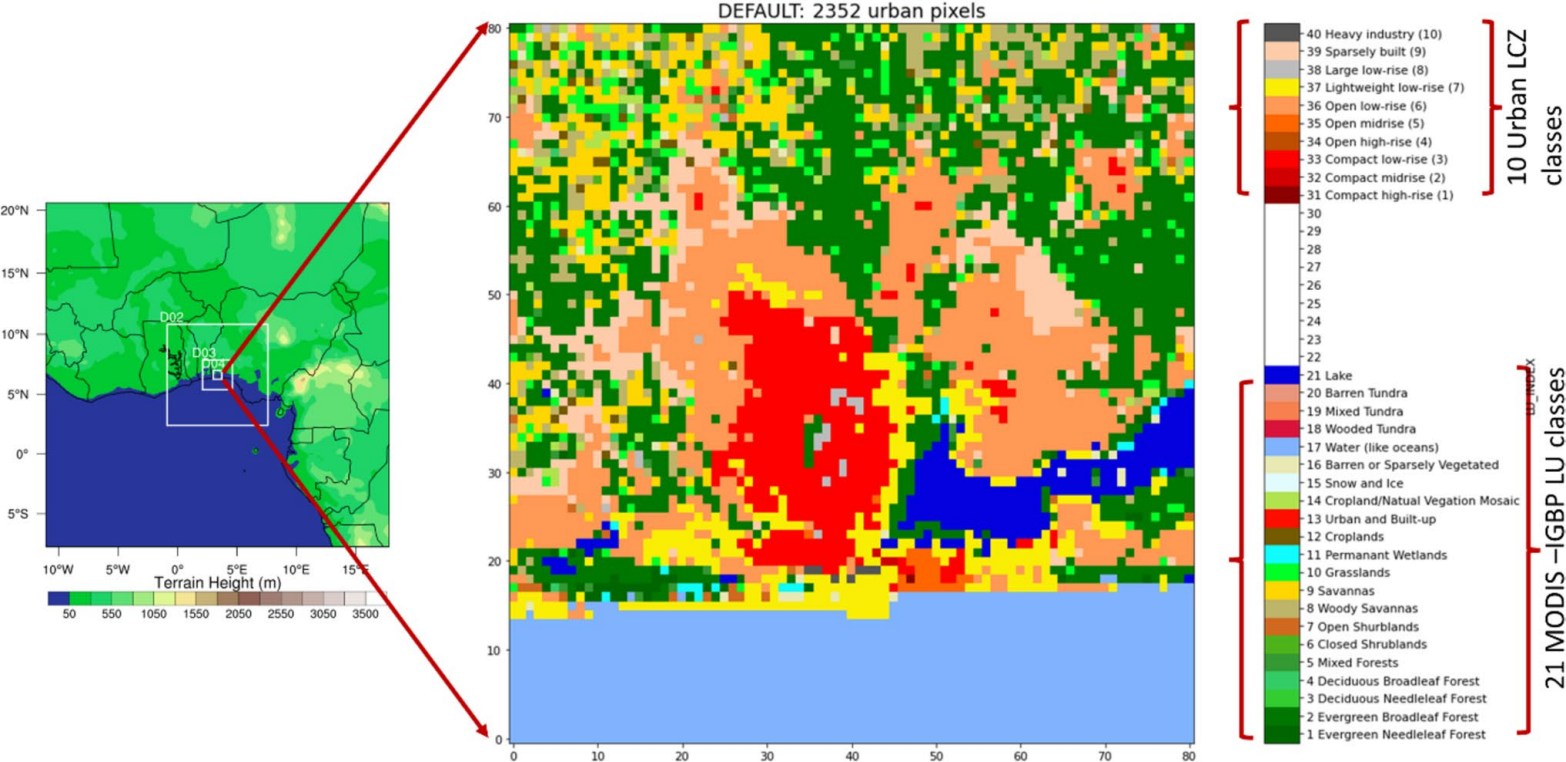
WRF urban configurations with four nested domains; the land cover map of the innermost domain is shown on the right where the urban pixels (2352) are replaced with detailed LCZ 10 built up classes

Similar to Bassett et al. (2020), WRF was configured with four nested domains set at 27-km, 9-km, 3-km, and 1-km resolutions (Fig. 3). The physical parameterization schemes were derived from the optimal combination of schemes used in previous studies over a tropical savanna climate (e.g., Bassett et al. 2020; Niyogi et al. 2020; Gbode et al. 2019). The cloud microphysics is used from WSM 6-class Graupel scheme (Lim & Hong 2010), surface layer is parameterized using Monin–Obukhov similarity scheme (Monin and Obukhov 1954), and land surface model considered in the study is the Noah land surface model (Liu et al. 2006). Boundary layer parameterization is from Bougeault and Lacarrère scheme (Bougeault and Lacarrere 1989), long wave radiation model used is the rapid radiative transfer model (RRTM: Mlawer et al. 1997), and for short wave radiation, Dudhia’s scheme (Dudhia 1989) is selected. The same physical parameterization schemes were maintained for all the simulations with the three UCM.

#### Model inputs

The WRF initial and boundary conditions were obtained from the 5th generation of the European Center for Medium-Range Weather Forecasting (ECMWF) reanalysis ERA5 data set. For outer domains, the default MODIS IGBP 21 land cover category data was used. However, for the innermost domain (d04), we implement the high-resolution LCZ map (Fig. 1a) for the urban land use (LU) category.

To incorporate the LCZ map into as part of the model geography setup, we also implemented Shuttle Radar Topography Mission (SRTM: Slater et al. 2006) in WPS. SRTM was delivered onboard the Space Shuttle Endeavor during an 11-day mission in February 2000 to create global most complete high-resolution (~ 90 m) topographic database.

As mentioned earlier, UCMs need information describing the 3D urban surfaces. These were sought for each LCZ from generic LCZ scheme. These values were based on the midpoints of the range of values suggested by Stewart and Oke (2012). Additionally, we obtained the averaged radiative and thermal properties for each LCZ from Stewart et al. (2012), Stewart et al. (2014), and Wouters et al. (2016) shown in the supplementary information (Table S2). Moreover, a more realistic anthropogenic heat flux was derived from the outputs of Surface Urban Energy and Water Balance Scheme (SUEWS: detailed in the SI) averaged over each LCZ within the domain.

#### Model design

Three distinct sets of model runs were made, each employing a different WRF-urban scheme. The first set “simA” employed the WRF_SLAB scheme. The second set “simB” utilized the WRF-BEP scheme. Lastly, “simC” with the WRF coupled with BEP + BEM scheme. This approach aimed to compare the model’s performance under varying WRF-urban schemes in others to find the optimum WRF-urban configuration for simulating meteorological variables for heat stress analysis. These simulations covered a 31-day period, specifically targeting a representative pre-monsoon period in March 2020, which is widely recognized for its heightened heat stress levels in southern Nigeria (Balogun et al. 2010). The prominence of heat stress during this month is primarily attributed to the influx of warm, moisture-laden maritime air from the Atlantic Ocean, leading to elevated humidity levels. The annual time series of mean daily air temperature for the year 2020 is shown in Fig. S3.

### Method of model evaluation

Observed air temperature (*T*_2_), relative humidity (RH), wind speed, and direction data on an hourly basis were obtained from the archive of the Nigerian Meteorological Agency (NiMet) and the Trans-African Hydro-Meteorological Observatory (TAHMO: van de Giesen et al. 2014). The data was collected for three locations within the Lagos metropolis: Lagos airport (LCZ8: 3.32°E, 6.58°N), University of Lagos (Unilag: LCZ 3: 3.39°E, 6.52°N), and Ikenne (LCZ 6: 3.70°E, 6.85°N) as shown in Fig. 1a. While we allowed 7 days of model spin-up, evaluations were performed for 24 days (March 8 to 31) for the hourly simulated data from simA, simB, and simC with observation data. The baseline comparison statistics included correlation coefficient (*R*), root mean square error (RMSE), and mean bias error (MBE) for performance assessment.

### Heat stress index and analysis

#### Heat stress index: humidex

A number of heat stress indices exist to quantify the risk of heat stress in humans (Havenith & Fiala 2016). In this study, humidex (Masterson and Richardson 1979) and wet bulb globe temperature (Botsford 1971) widely used in heat stress assessment in coastal cities were comparatively adopted to examine the magnitude and spatial variability of heat stress due to differential landscape features and background climate in a megacity like Lagos. Humidex is a measure of the combined effect of temperature and humidity on the human body. It is a term designed by the Canadian meteorologist to describe the perceived temperature in hot and humid weather conditions. Although humidex is unitless, it is sometimes estimated in degrees Celsius because it is a measure of a person’s perceived temperature. As the air temperature and humidity increase, the human body’s ability to transport metabolic heat through evaporative cooling reduces (Sherwood & Huber 2010).

1 $${\text{Humidex}}={T}_{2}+h$$

where “${T}_{2}$” is the actual 2-m air temperature in degrees Celsius and “$h$” is a value that depends on the relative humidity of the air defined as

2 $$h=\left(0.5555\right) \times \left(e-10\right)$$

where “$e$” is the vapor pressure of the air in kilopascals (kPa). The vapor pressure can be calculated using the following formula:

3 $$e=6.11 \times \left({10}^{\frac{7.5{T}_{2}}{237.7+T2}}\right) \times \frac{RH}{100}$$

where “$RH$” is the relative humidity in percentage.

Once the value of “$h$” has been calculated, it is then added to the actual air temperature to obtain the humidex value.

The higher the humidex, the harder it is for sweat to evaporate from the skin, which makes it feel hotter than it actually is. Typically, humidex is used as an indicator of outdoor thermal comfort; however, a study by Rana et al. (2013) shows that it is also a good indicator of indoor thermal comfort. The index was used in an epidemiologic study of mortality during the summer 2003 heat wave in Europe (Conti et al. 2005). Humidex scale typically ranges from 0 to 50 (Table 1), with values above 30 indicating conditions that may be uncomfortable for some people and values above 40 indicating conditions that can be dangerous for anyone spending time outdoors. We, however, provided a summarized linear relationship between humidex and WBGT (Fig. S5), estimated with the best performing WRF-urban scheme, in the supplementary information to further justify our humidex selection. Our analysis revealed a 0.8 correlation coefficient between the two indices. This agrees with the results of Heidari et al. (2016), who concluded that humidex can be applied as an appropriate substitute for the WBGT index.

**Table 1:** Classification of heat indices and heat risk conditions

Classification of heat condition	Humidex (°C)	General effect on people no
No risk	> 29	No risk to population groups
Very warm	30.00–38.99	Fatigue POSSIBLE with prolonged exposure and/or physical activity
Hot	39.00–41.99	Sunstroke, heat cramps, or heat exhaustion LIKELY and heat stroke POSSIBLE with prolonged exposure and/or physical activity
Very hot	42.00–44.99	Sunstroke, heat cramps, or heat exhaustion POSSIBLE with prolonged exposure and/or physical activity
Extremely hot	> 45	Heat/sunstroke HIGHLY LIKELY with continued exposure

Source: adapted from (Masterton and Richardson, 1979; Kotharkar et al. 2021)

For the rest of this paper, our heat stress analysis is based on humidex with focus on a particular heat wave period derived based on 3–4 consecutive days with maximum temperature greater than the climatological mean (Frich et al. 2002). For Lagos, the climatological mean temperature is 28.4 °C for the month of March (1991–2020: Climate Data Online). Therefore, our heat stress analysis is based on heatwave period that occurred between 16 and 20 March 2020. The methodological framework is summarized shown in Fig. 2.

#### The pattern of heat stress and urban impact on heat stress

To investigate the intra-urban variation in heat stress, we initially analyzed the spatial and diurnal patterns of heat stress during a representative heat wave period. Furthermore, we computed the frequency of occurrence for each heat stress category and compared them within each urban LCZs.

To assess the urban impact, we conducted another two sets of simulations using the top-performing WRF-urban scheme: the first simulation (simD—with urban) and second scenario (simE—without urban). In this scenario, urban pixels were replaced with vegetation to create a comparative baseline. This was run for a heatwave period that occurred between 16 and 20 March 2020. For each simulation, we computed humidex (HI) values as indicators of heat stress.

The difference between the two simulations was quantified as follows:

4 $$\Delta HI = {HI}_{{\text{urban}}}- { HI}_{{\text{no}}-{\text{urban}}}$$

where $\Delta HI$ is the urban influence on heat stress pattern, ${HI}_{{\text{urban}}}$ is the heat stress estimated from the meteorological parameters from simD, and ${HI}_{{\text{no}}-{\text{urban}}}$ is that of simE.

## Results and discussion

### Model evaluations

#### Comparisons of UCM simulated air temperature and relative humidity with ground observation data

**Fig. 4:**
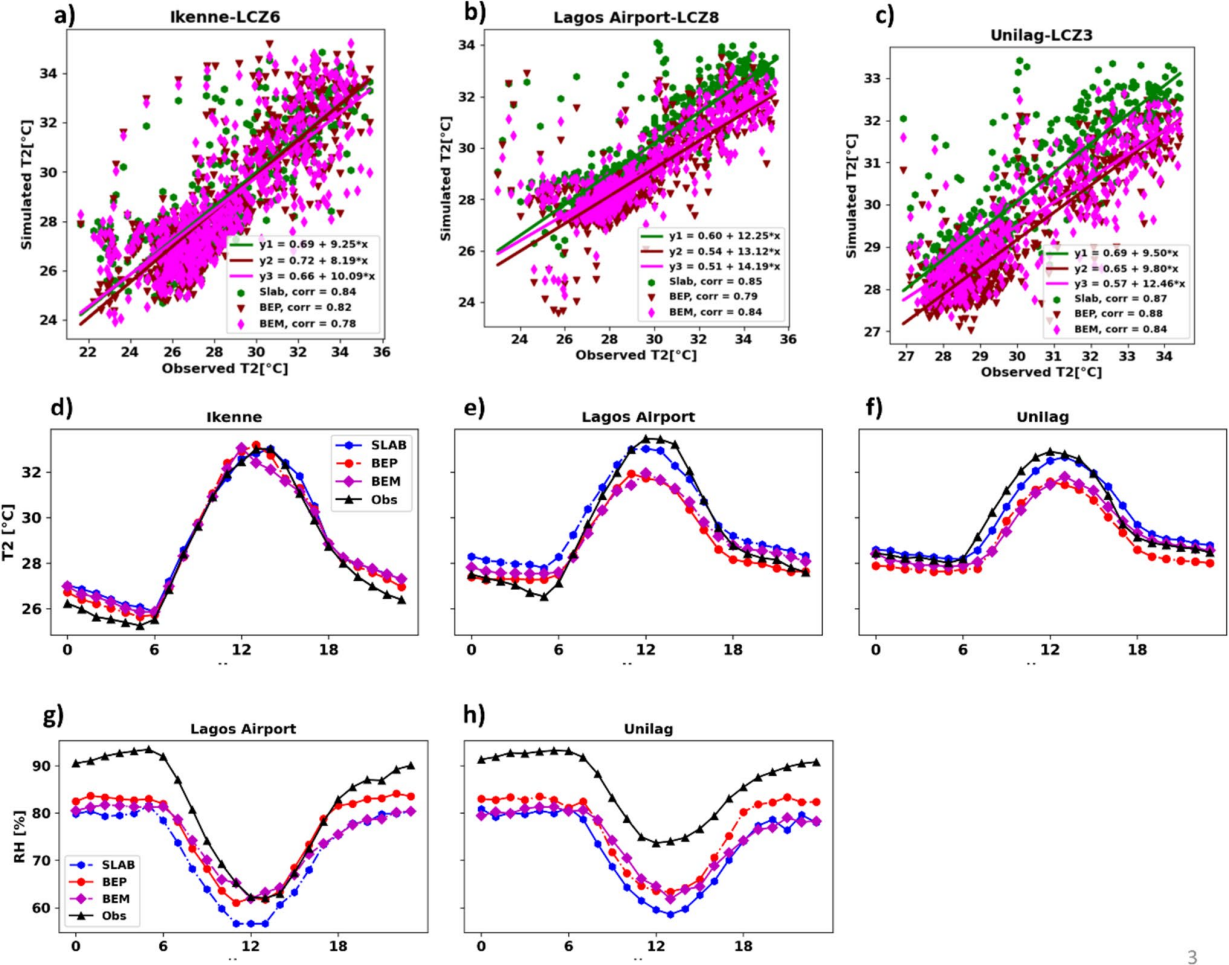
Scatter plot showing the linear relation between the three UCM predictions and simulated *T*_2_ (°C) for the three locations. The middle and the lower panel shows the comparison of mean diurnal pattern of 2-m air temperature (middle panel, **d**–**f**) and relative humidity (%) (lower panel, **g**, **h**) of simulations with three different UCMs vs observations. Note: the diurnal pattern of RH is not plotted over Ikenne due to the absence of observation data

The mean diurnal pattern and the linear relationship between simulated and observed of air temperature (*T*_2_) are illustrated in Fig. 4. Generally, we observed that the models all captured the diurnal pattern of *T*_2_ albeit with some slight underestimation and overestimations. Specifically, at Ikenne station, classified as LCZ C (open arrangement of short bushes), the models slightly overestimated nighttime *T*_2_. One possible reason for the *T*_2_ biases in the models could be the misrepresentation of the exact measurement location within the model grid at 1 km. While at Ikenne station classified as LCZ C (bush), a rural area with vegetation, the model grid may have incorporated some urban features that could influence the nighttime *T*_2_. This misrepresentation might have caused the model to predict slightly higher *T*_2_ values than observed, as it failed to fully capture the cooling effects of evapotranspiration of the vegetative surface. Similarly, at Lagos airport location, which is classified as LCZ 8 (open arrangement of large low-rise buildings), even though some large open-rise buildings are present, the exact measurement location is on low grass with the nearest building at least 100 m (standard meteorological station). Thus, the model assumes a land cover mostly paved (which is an attribute of LCZ 8) to the whole grid (1 km^2^) and thus the slight warm bias at nighttime due to the release of ground heat flux. On the contrary, we noted that the models underpredict the *T*_2_ during the day in Unilag and Ikenne locations. This could be due to the models accounting for the cold advection of sea breeze at this time. Hence, the cold advection systematically lowers the air temperature. Besides, the two locations are closer to the coast than Ikenne stations far inland to the northeast of the study area. The thermal gradient caused by the cool air advection has recently been noted by Wermter et al. (2022), who observed that latitudinal thermal gradient is connected to the occurrence of sea breeze events. Again, shading and reflections are two important factors that could probably lead to underestimations of daytime *T*_2_.

In Ikenne and Unilag locations, we noted that the SLAB model performs better than BEP and BEP + BEM models because the two multilayer models underestimate the daytime *T*_2_. The pattern of SLAB model seems to be closer to observation in these two locations because the SLAB model does not account of urban landscape heterogeneity and anthropogenic heat fluxes and assumes higher values for surface thermal properties. In the evening hours (from 1800 h), we noted that the lowest *T*_2_ values were recorded by the BEP model, particularly in high density urban locations (Lagos airport, LCZ8, and Unilag, LCZ 3). This suggests that heat is dissipated rapidly in the BEP model, whereas the BEP + BEM model retains more heat because of the inclusion of anthropogenic heat. These findings corroborate the results obtained by Ribeiro et al. (2021), who also found that BEP predicted the lowest *T*_2_ values during morning and evening hours in Barcelona when compared to the SLAB scheme and BEP + BEM. Quantitatively assessing the biases, we found that the SLAB model exhibited a general warm bias, with a mean bias error (MBE) of 0.34 °C. In contrast, both the BEP and BEP + BEM models showed cold biases, with MBEs of − 0.34 °C and − 0.15 °C, respectively. These results were to be expected since the multilayer schemes (BEP and BEP + BEM) take into account urban landscape heterogeneity, cooling effects of urban greenery, evapotranspiration, and the influence of sea breeze. In contrast, the SLAB model lacks urban area discretization, which contributes to the warm bias observed in its predictions.

In terms of linear relationship between observed and simulated *T*_2_, all three UCMs show a good correlation (> 0.7) in all the locations. However, the SLAB model had the highest correlation value, with an average value of *R* = 0.85, while the multilayer models of BEP and BEP + BEM had an average value of *R* = 0.83 in the three locations. The SLAB model had the lowest root mean square error (RMSE = 1.47 °C), followed by BEP + BEM (RMSE = 1.59 °C), and the worst performance was observed with the BEP model (RMSE = 1.63 °C). Generally, we noted that the range of RMSEs for simulated *T*_2_ was similar to that reported by Bassett et al. (2020). Additionally, our ensemble model demonstrates a significantly improved bias, with 2.13 °C bias during the day, in contrast to Bassett et al. (2020), who reported a bias of 5.18 °C. The heat maps are displayed in the supplementary information (Fig. S6).

For RH, the mean diurnal pattern over Lagos airport and Unilag is illustrated in Fig. 4g, h. Generally, the diurnal trend of RH is underestimated by the three UCMs. The largest underestimation is noted in the SLAB model, especially in Unilag location. Again, this could be due to the effects of the sea breeze and the evaporation rate from water bodies that cannot be captured by the models as Unilag is closer to the coast. The BEP model seems to perform best in both locations. SLAB presents a wet bias of 13% followed by BEP + BEM and BEP models with a wet bias of 11% and 9%, respectively. Difficulty in simulating relative humidity can be attributed to factors such as soil moisture initialization and the land surface model used in WRF (Jain et al. 2017). Similar patterns of WRF biases in relative humidity in comparable climates were noted by Mohan and Bhati (2011), Singh et al. (2022), and Bilang et al. (2022).

#### UCM simulations of the spatial pattern of air temperature and wind speed

**Fig. 5:**
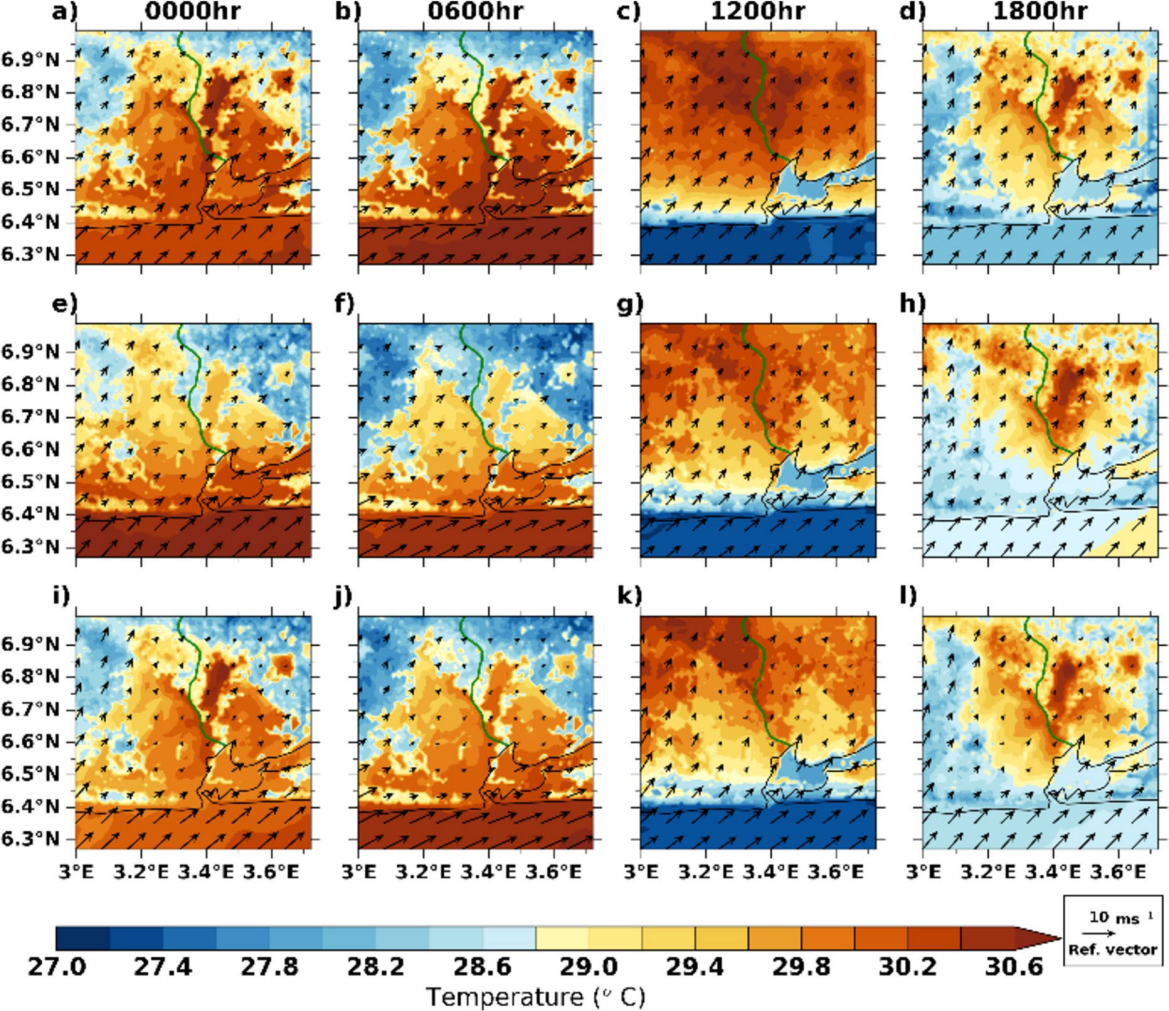
Averaged wind fields (10 m) and air temperature at 00 h, 06 h, 12 h, and 18 h (UTC) in the month of March 2020: SLAB (top panel), BEP (central panel), and BEP + BEM (bottom panel)

Figure 5 illustrates the spatial pattern of *T*_2_ and windspeed simulations at 0000 h, 0600 h, 1200 h, and 1800 h SLAB, BEP, and BEP + BEM. At nighttime and early morning (0000 and 0600 h), the SLAB model predicts *T*_2_ and wind speed in a rather more homogeneous pattern across the domain, which could probably be due to the fact that the SLAB model does not allow the discretization attribute of urban areas into different land cover types whereas the impact of urban landscape heterogeneity is evident in BEP and BEP + BEM simulations. BEP and BEP + BEM take the 10 urban classes of the LCZ into consideration.

Similar to 0600 h, SLAB predicts homogeneous *T*_2_ patterns during daytime (1200 h), whereas intra-urban variations of *T*_2_ patterns are evident in multilayer schemes, with compact urbanized areas having higher *T*_2_. Additionally, we found that highly urbanized areas have lower air temperatures at this time in comparison to the suburban/rural areas at this time. This is particularly evident in BEP and BEP + BEM models, whereas the pattern is so obvious in the SLAB model as illustrated in Fig. 5c, g, k. First, this might be due to advection of cool sea breeze at this time of the day. This pattern of lower urban temperature at this time penetrates deeper in the BEP + BEM model (up to 6.7°N) than the BEP model. This corroborates the findings of Acero et al. (2013) who noted urban cool island in northern Spain due to the sea breeze effects. While the direct influence of sea breeze on heat stress patterns in sub-Saharan African cities is yet to be fully understood, research in tropical cities suggests that sea breeze can modify urban temperature patterns and shift the center of the heat island circulation further inland (Freitas et al. 2007; Mughal et al. 2019). Specifically for Lagos, Bassett et al. (2020) found that the heat island is advected to the rural northeastern axis. Secondly, the observed pattern of air temperature might also be attributed to the inclusion of shading and reflections in multilayer models. As tall towers increase shading in the core urban canyon, radiation trapping is reduced. Generally, the high temperature in non-urban areas than the core urban areas, especially those closer to the coast, indicates the possibility of urban cool islands in some areas of Lagos during the day which could significantly influence the pattern of heat stress.

Moreover, it is also clear that, in spite of the closeness of BEP and BEP + BEM in terms of physical configurations, BEP + BEM presents similar *T*_2_ patterns to SLAB than to BEP at 1800 h. This suggests that heat is lost more rapidly as the incoming solar radiation intensity decreases in BEP whereas BEP + BEM retains more heat due to it accounting for more anthropogenic heat fluxes. In fact, *T*_2_ by BEP are the lowest at nighttime in comparison to the other two schemes.

For the airflow within the domain, since BEP and BEP + BEM consider building structures and street canyons’ influence on airflow, they aimed a more realistic flow pattern across the urban landscape unlike SLAB model which predicts a homogeneous pattern of airflow across the domain. BEP + BEM predicts weaker winds than the other schemes during the whole period, especially during the daytime, which could be attributed to the implementation of drag coefficient in BEP + BEM (for more detailed evaluation results regarding wind speed and direction, see supplementary material).

To determine the optimal model configuration, the root mean square errors (RMSEs) of three UCMs were normalized for temperature, wind speed, and relative humidity. The results are presented in Fig. S12, which can be found in the supplementary information. The BEP model had the lowest normalized root mean square error (NRMSE) of approximately 0.28, followed by the BEP + BEM model with an NRMSE of 0.3 and the SLAB model with an NRMSE of 0.38. These findings suggest that the BEP model is the most accurate in local meteorological variables for heat stress analysis.

#### Impact of model bias on the simulated heat stress pattern

**Fig. 6:**
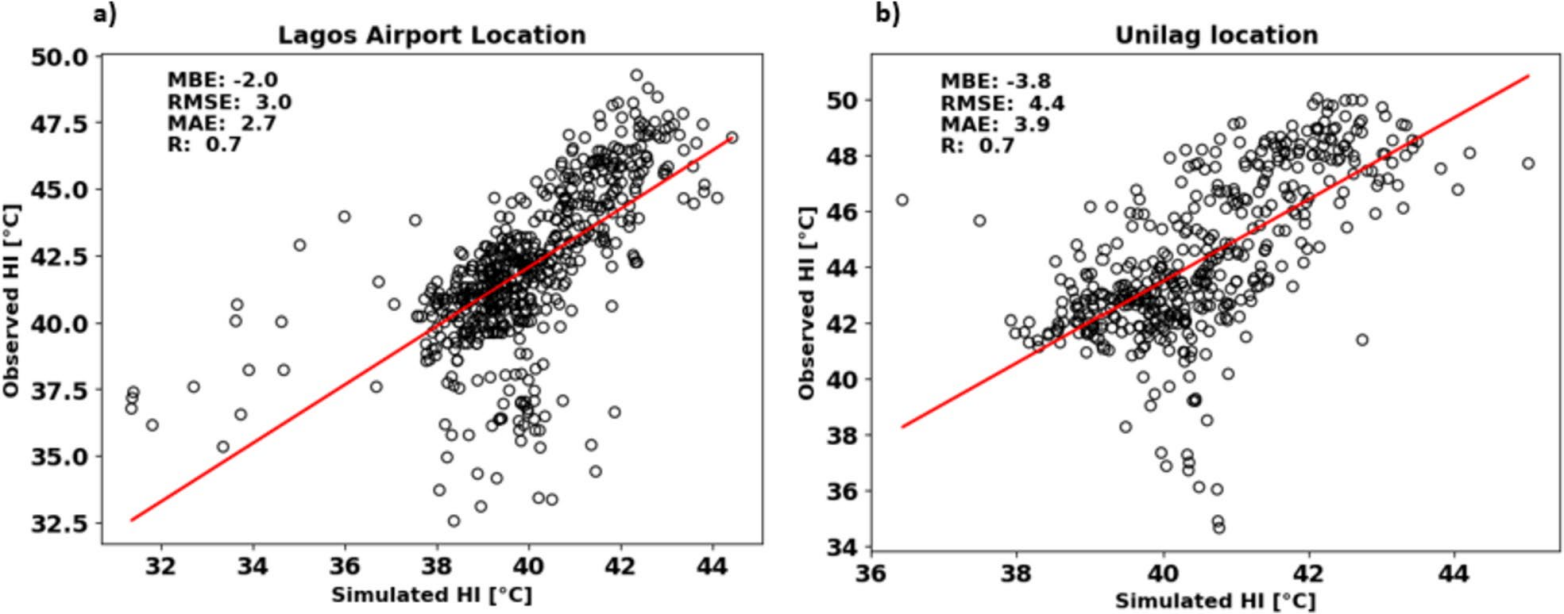
Scatter plots comparing the simulated and observed humidex (HI) at **a** Lagos airport and **b** Unilag locations

Before delving into the analysis of spatial and temporal heat stress patterns, this section addresses the impact of model bias on estimated heat stress. Figure 6 illustrates the scatter plots comparing observed and simulated humidex (HI) values at two locations (Lagos airport—LCZ8 and Unilag location—LCZ3) with complete air temperature and relative humidity data. Overall, we observed that the model tends to underestimate heat stress in both locations. Specifically, the MBE for Lagos airport and Unilag locations were − 2.0 °C and − 3.8 °C, respectively. The RMSE was 3.0 °C for Lagos airport and 4.4 °C for Unilag location. The MAE for Lagos airport was 2.7 °C, while for Unilag location it was 3.9 °C. Despite these biases, there is a strong positive correlation between the observed and simulated humidex values in both locations (*R* = 0.7). The primary contributing factor to the bias in predicted heat stress appears to be the model’s difficulty in accurately forecasting relative humidity (RH), which tends to be underpredicted.

### Heat stress analysis: humidex

#### Spatiotemporal assessment of heat stress during heat wave period over Lagos

**Fig. 7:**
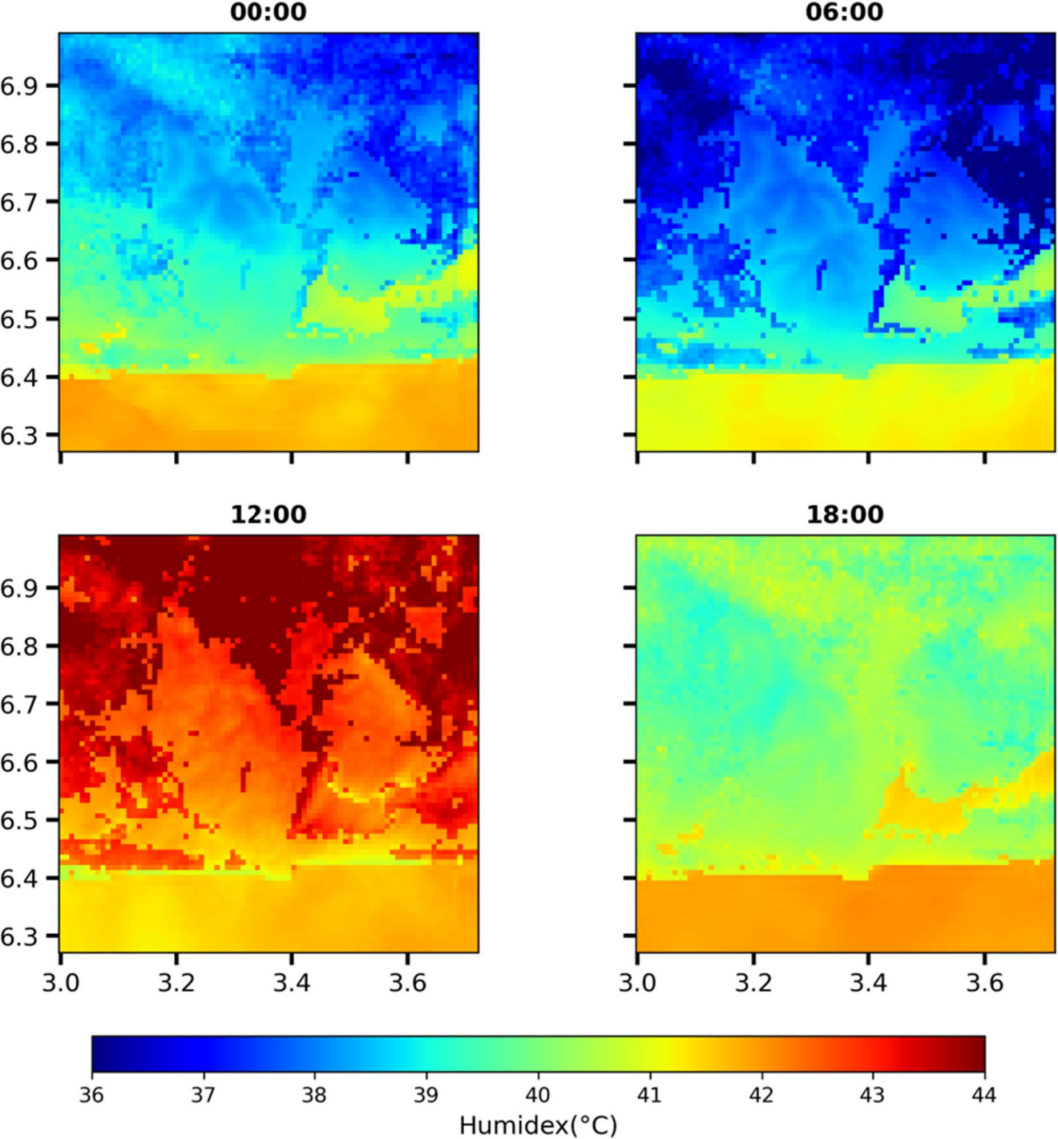
Spatially averaged humidex values at different times of the day (00:00, 06:00, 12:00, and 18:00)

The spatial variation of the heat stress index—humidex, estimated using the simulated output of the BEP model—the best performing WRF urban scheme at different times of the day, is presented in Fig. 7. There is evident disparity in the heat stress values observed within the core urban and suburban/rural areas at night at all times. Expectedly, the highly urbanized areas are clearly distinguished as hot spots during the nighttime (00:00) and early morning (06:00). At nighttime, mostly “hot” conditions predominate (humidex = 40–42 °C) the core urban areas and “very warm” conditions in the suburban and rural areas. High heat stress in the core urban areas at this time is due to the combined effect of nighttime urban warming (UHI) and high relative humidity in the urban core. As expected, urban air temperatures are considerably higher than corresponding air temperatures in the suburban and rural areas at this time (Ojeh et al. 2016). In addition to increased air temperatures, Lagos urban areas present high relative humidity (RH > 84%) during this period. The suburban and the rural areas generally showed lower humidex values with “very warm” heat stress conditions, which is largely due to high pervious surfaces, higher vegetation cover, high sky view factor, and all other attributes of suburban and rural areas. However, since the relative humidity in these areas is generally higher than in the core urban areas due to evapotranspiration. This reveals that the lower heat stress conditions are due to the lower air temperature in rural areas at night. At 06:00, the humidex values decrease significantly in both urban and suburban/rural areas to remain in “very warm condition.” However, there are still noticeable differences in the humidex conditions between urban and rural areas.

Unexpectedly, “very hot” conditions were observed in less urbanized areas and “hot” conditions were observed in the core urban areas at noon (12:00). Theoretically, this could be attributed to the stronger sea breeze effect. At this time, the advection of cold sea breeze lowers the air temperature in the urban core adjacent to the coast which couples with the lower humidity values observed during the day to yield lower humidex values than the suburban areas with higher temperature and high humidity exacerbated by evapotranspiration. Generally, as this is a pre-monsoon season, winds are south-westerly with increased speed, advecting the heat further inland from the urban core to the north-eastern axis of the domain which are predominantly non-urban areas. Additionally, we can also put a case for shading effect of urban tall structures to be a contributing factor for the reduced heat stress at this time.

At evening hours (18:00), the less urbanized areas lose more heat quickly due to factors such as vegetation and open spaces, which can allow heat to dissipate more easily whereas the core urban areas retain heat due to the urban density, resulting in all areas being under “very warm” conditions at this time. In general, the higher nighttime heat stress observed in highly urbanized areas can be attributed to the UHI effect. On the other hand, the lower heat stress during the day may be influenced by the significant role of the cold sea breeze and/or the shading effect from tall urban buildings. During this period in March, the high moisture content in the air is mainly due to the advection of moisture-laden maritime air from the Atlantic Ocean, which is typical during a pre-monsoon season (Lélé et al. 2015; Fitzpatrick 2016). The presence of this high moisture content exacerbates heat stress and leads to an increased level of discomfort.

**Fig. 8:**
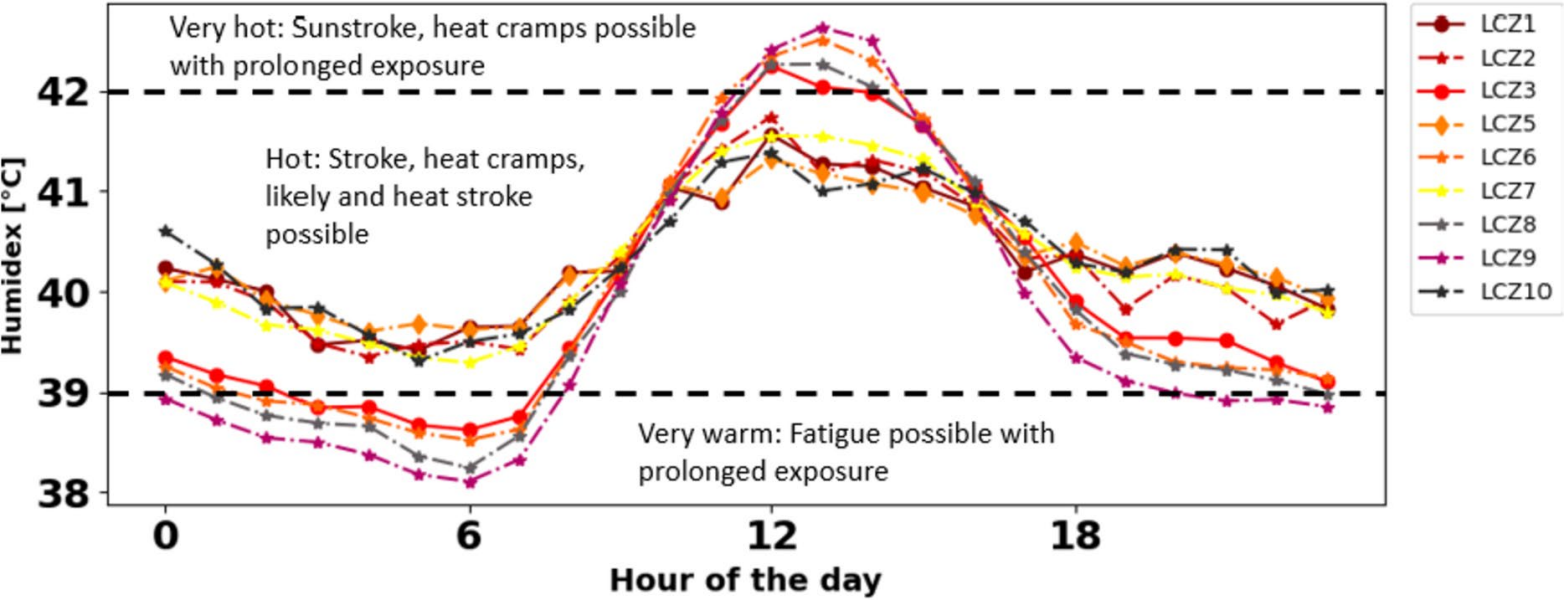
Mean diurnal pattern of heat stress in urbanized LCZs

Specifically, the mean diurnal variations of heat stress conditions in dominant LCZs are illustrated in Fig. 8. It is observed that during the night, LCZs 7, 1, 2, 5, and 10 experienced “hot” conditions, while suburban LCZs 6 and 9 experienced between “very warm” and “hot” conditions, whereas at noon suburban LCZs 6 and 9 experienced “very hot” conditions. This revealed that core urban areas experience higher levels of heat stress at night due to the combined effect of urban warming and higher relative humidity in the urban core. We also found that air temperatures in urban areas are higher than those in suburban areas, and Lagos urban areas have high relative humidity during this period. The high moisture content in the air exacerbates heat stress and increases discomfort levels.

#### Impact of urban morphology on the spatial variation of heat stress

**Fig. 9:**
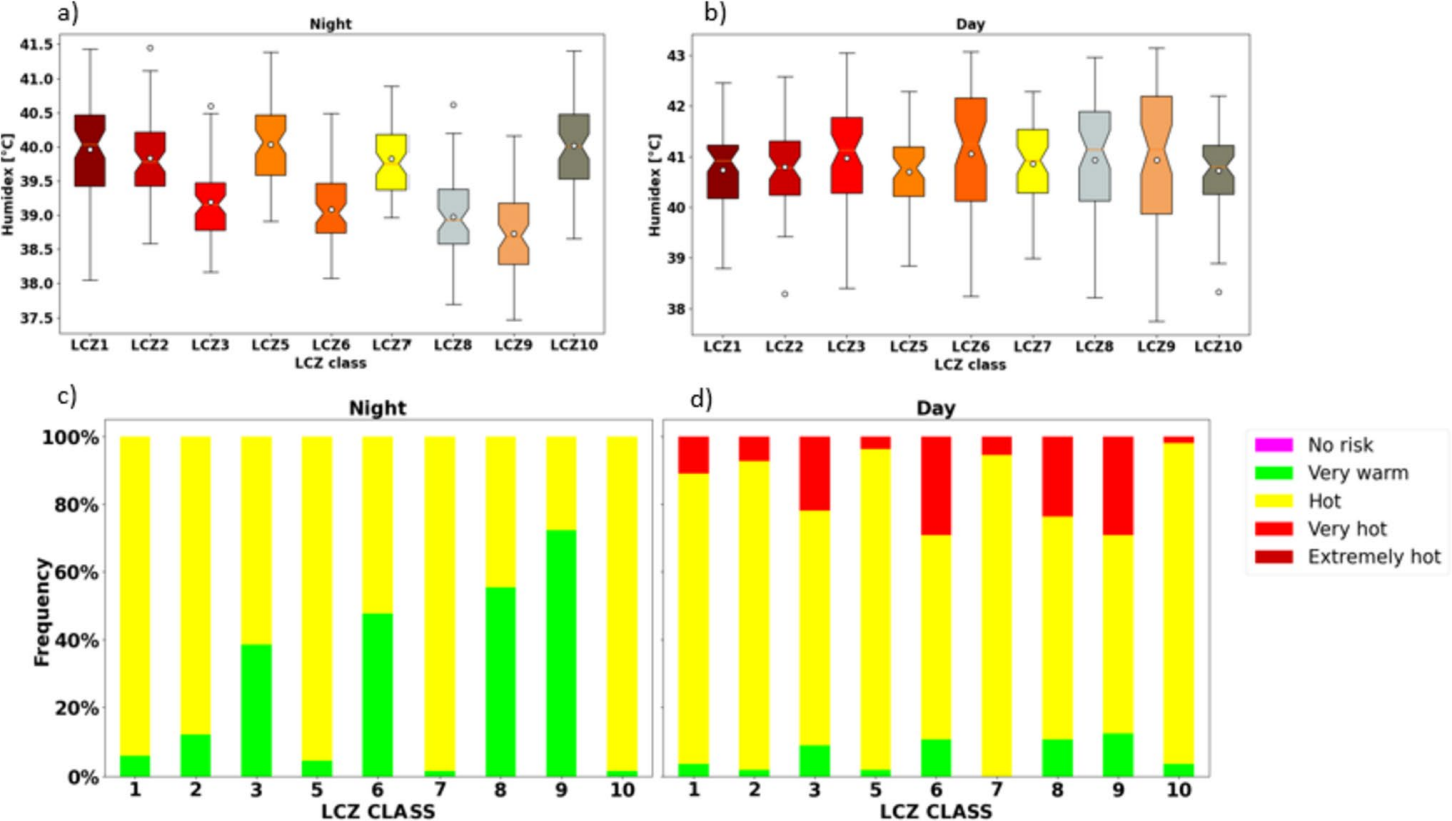
**a**, **b** Box plot showing the distribution of mean humidex values for each urban LCZs. **c**, **d** Number of hours with “very warm,” “hot,” and “very hot” heat stress conditions with respect to each urban LCZs during heatwave period (March 16–20, 2020) during the night hours (18:00 to 06:00) and day (07:00 to 17:00)

The LCZ urban categorizations are based on the idea that urban areas have unique physical characteristics that influence their energy and water balance and, therefore, have distinct effects on their local climate (Stewart & Oke 2012). In this context, Fig. 9a, b shows distinct variability in the humidex values within the urbanized LCZs. During the night, we generally observed lower humidex values in highly vegetated and sparsely built urban areas LCZ 6 and LCZ 9 as the median values are skewed towards lower humidex values. Conversely, low vegetated and highly urbanized LCZs 1, 2, 5, and 10 have higher humidex with maximum values of 41.5 °C, 41.0 °C, 40.0 °C, and 40 °C, respectively. Surprisingly, we noted high humidex values in LCZ 7 which is a lightweight low-rise building. In Lagos, these areas are often classified as informal settlements which are densely built and highly populated. Essentially, this LCZ consists of areas with hard-packed lightweight building materials such as corrugated metal, possessing high thermal properties that can retain more heat and contribute to thermal stress at night.

At daytime, unlike at nighttime, heat stress is reduced within the core urban areas. The location of the particular LCZ, as well as the urban morphology, comes into play. This variation can be attributed to the interplay of the location of the specific LCZs and the morphological characteristics. Notably, highly urbanized areas in LCZs 1, 2, 5, and 10 are in closer proximity to the coast compared to LCZs 3, 8, and suburban zones 6 and 9. As a result, they may experience more pronounced cooling effects from the sea breeze, influencing their exposure to heat stress. Additionally, highly compact and tall LCZs (e.g., LCZ 1, 2, and 5) consist of tall structures that could potentially diminish radiation retention through shading, thereby contributing to cooler temperatures within the urban core. Moreover, the highly impervious nature of these LCZs implies reduced relative humidity, which further contributes to the comparatively lower heat stress observed in the urban core, in contrast to suburban LCZs. Overall, our results indicate the impact of different urban landscape characteristics, however with a stronger effect on the heat stress at night. This finding corroborates previous studies on heat stress (e.g., Chakraborty et al. 2022), who found that heat stress in European cities are some fraction lower than suburban and rural areas.

Figure 9c, d illustrates the frequency of times (hours) associated with different levels of heat stress. In Fig. 9c, the areas with the highest frequency of “hot” conditions during the night are LCZ 7 and 10, followed by LCZ 1, 2, and 5. Exposure to “hot” heat stress conditions during the night can lead to heat-related health issues such as heat cramps or heat exhaustion, and with prolonged exposure, heat stroke is possible. Our preliminary analysis (results not shown) and corroborated by Badmos et al. (2020) and Simon et al. (2013) shows that a significant proportion of Lagos residents live in these areas and therefore are more likely to be exposed to high levels of heat stress during the night. In addition, the people that live in these areas are socioeconomically disadvantaged without basic electricity and adequate sanitation (Badmos et al. 2020) and therefore have higher risk of heat-related risks. Unlike LCZ 7, suburban LCZ 6 and 9 have a larger proportion of “very warm” hours during the night, which can also in some cases cause fatigue with prolonged exposure.

During the day in Fig. 9d, all LCZs experience some degree of “very hot” conditions, with LCZs 6, 8, and 10 being the most affected, followed by highly urbanized LCZs. As discussed in previous sections, even though LCZ 6 and 9 have more vegetation and are sparsely built, while the urban core LCZs (1, 2, 3, and 5) are densely built with less vegetation, they still experience similar levels of heat stress during the day (from 7:00 to 17:00). This suggests that the reduced radiation trapping and/or cool sea breeze advection may be the primary factors moderating the level of heat stress during the day.

**Fig. 10:**
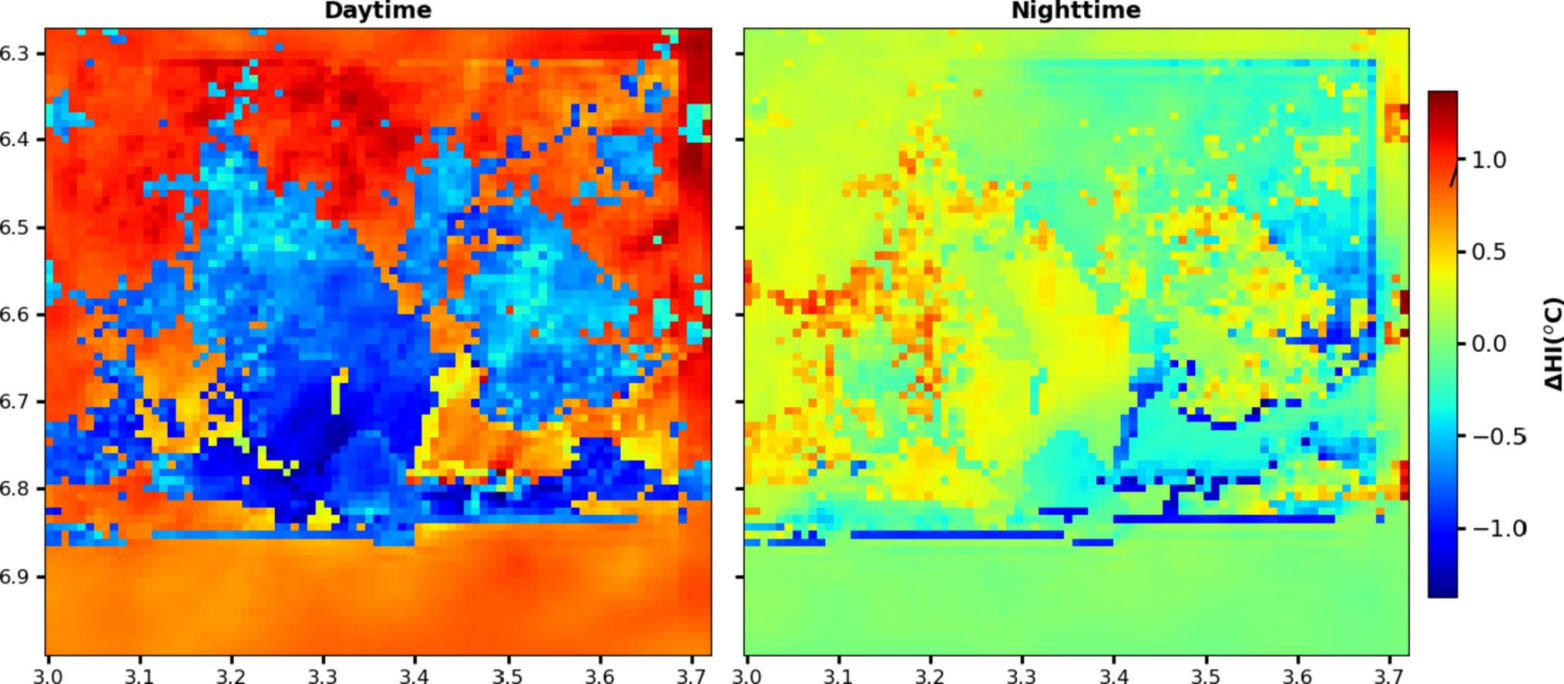
Spatial pattern of urbanization impacts on the heat stress during the day and at nighttime

Further, we noted a close match between humidex values and the urban morphological features. High humidex values were found in high compact and industrialized urban areas than low, less compact and highly vegetated urban areas. This implies that the urban morphological features also play a core role in the heat stress pattern in Lagos. Figure 10 depicts the contrasting effects of urbanization on heat stress patterns during daytime (07:00 to 17:00) and nighttime (18:00 to 06:00). In the daytime, a noticeable reduction in heat stress is evident, especially in highly urbanized regions. However, the opposite trend is observed during nighttime, with urbanization contributing to heightened heat stress.

The increased heat stress due to urbanization is most pronounced in suburban areas (LCZ6 and 9). This pattern could be attributed to the UHI effect and the urban cool island (UCI) effect. During daytime, the interplay of shading and decreased relative humidity in urban areas yields a cooling effect, leading to diminished heat stress. Conversely, during the night, the urban influence becomes notable. Heat released in urban areas elevates air temperatures, intensified by increased humidity, resulting in high heat stress.

These divergent dynamics showcase the intricate interrelationship between urbanization, temperature fluctuations, and humidity variations, emphasizing the pivotal role of UHI and UCI phenomena in shaping the urban heat stress landscape.

## Key findings and conclusions

The assessment of heat stress in highly urbanized humid tropical regions is limited by the lack of high-resolution weather data, presenting a significant research gap. In this study, we aimed to address this limitation by building on previous WRF-urban study in Lagos using an improved urban canopy representation of the LCZ scheme to investigate the effectiveness of WRF-urban schemes, in simulating local meteorological data for urban climate studies in sub-Saharan Africa and to understand the spatial pattern of heat stress within the urban area. We found that (1) the locally generated LCZ for Lagos, incorporating local knowledge, offers a more accurate representation of informal urban areas typical of the sub-Saharan Africa context compared to the global LCZ map. (2) The multilayer urban canopy scheme of WRF-BEP performs best in simulating local basic meteorological data necessary for heat stress analysis. This is achieved with the lowest NRMSE (25%) compared to 20% by WRF BEP + BEM. (3) The analysis of model bias in simulating heat stress patterns indicates a consistent underestimation of humidex values. Despite this bias, a strong positive correlation between observed and simulated humidex values persists in both locations. The primary contributing factor to the underestimation appears to be the model’s difficulty in accurately forecasting relative humidity, which consistently tends to be underpredicted. And (4) during nighttime, informal urban areas in LCZ 7 and highly urbanized LCZs 1, 2, 3, and 5 exhibit higher heat stress conditions than suburban areas. Conversely, during the daytime, suburban LCZs 6 and 9 experience the highest frequency of heat stress hours.

Notwithstanding, it is essential to acknowledge certain limitations. Firstly, we acknowledge the presence of model biases in predicting heat stress values, primarily attributed to challenges in accurately forecasting relative humidity. In this study, we assume that these biases are uniform across the entire domain, enabling inter-urban heat stress comparisons. However, we highlight the need for improvements in the model’s performance and advocate for additional ground truth data to enhance model evaluation. Secondly, our reliance on the LCZ scheme for intra-urban classification in our WRF simulation introduces a limitation. Despite its significance in global UHI studies, the LCZ scheme offers a simplified classification of urban land cover and land use types but fails to capture certain informal urban areas in sub-Saharan Africa, such as coastal informal settlements like those in the Makoko area of Lagos (Simon et al. 2013). To address this limitation, we recommend future microscale research involving fieldwork or ground-truthing to accurately map out these intricate areas.

Moreover, in our heat stress assessment, further research is needed to specifically separate the impacts of urbanization and the background climates on the heat stress patterns. Additionally, while heat stress hazard analysis is an important aspect of heat risk assessment, a more comprehensive understanding of vulnerability and exposure to heat stress is necessary.

In general, the results of this study have practical implications for urban planning and design in Lagos. Identifying areas experiencing higher heat stress can guide targeted interventions to mitigate the impacts in hot spots. Implementing measures such as co-designing incorporating green spaces, increasing tree canopy cover, and promoting cool roof initiatives can help alleviate heat stress in highly urbanized areas, potentially reducing the advected heat to the suburban/rural areas in the northeast.

### Supplementary Information

Below is the link to the electronic supplementary material.

Supplementary file1 (DOCX 1264 KB)

## Data Availability

Data will be made available on request.
