# Supplementary material for: An assessment of WRF-urban schemes in simulating local meteorology for heat stress analysis in a tropical sub-Saharan African city, Lagos, Nigeria
Source: Int J Biometeorol. 2024 Feb 16;68(5):811–28. doi: 10.1007/s00484-024-02627-3 (PMC11058602; doi:10.1007/s00484-024-02627-3)
Supplement: Supplementary file 1 — Supplementary file1 (DOCX 1264 KB) [file 484_2024_2627_MOESM1_ESM.docx]

**An assessment of WRF-Urban schemes in simulating local meteorology for heat stress analysis in a tropical Sub-Saharan African city, Lagos, Nigeria**

**Introduction.**

This supplementary information provides additional details about the methods used, the selected study period, and the heat stress index employed in the study. Furthermore, we present information regarding the pattern of the Urban Heat Island (UHI) and include additional figures that showcase model evaluations.


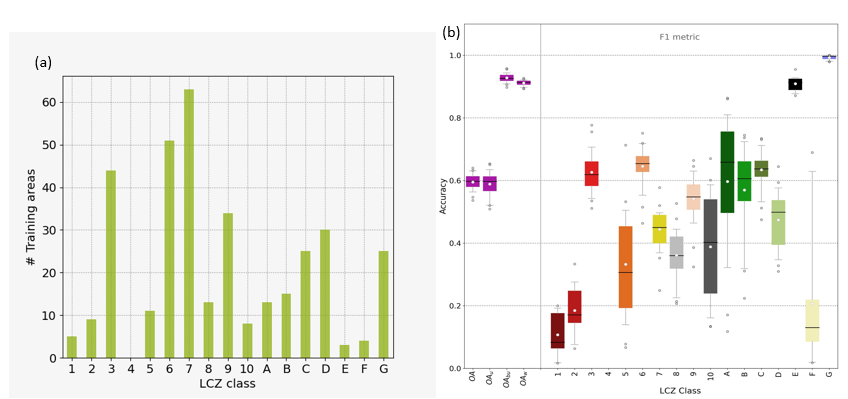


Fig. S1- Proportion of TAs selected per Local Climate Zone and b) the accuracy level of LCZ class. Note: OA is the overall accuracy.

1. **Comparison of LCZ map derived from locally derived TAs and global LCZ map**


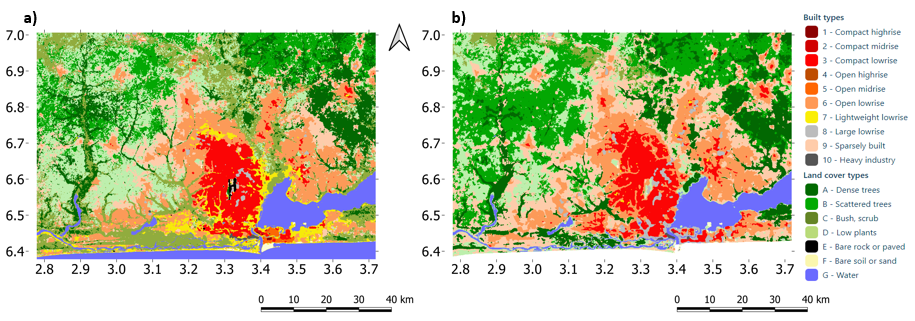


Fig. S2. Visual comparison of Lagos LCZ maps a) generated from locally deribed Tas and b) global LCZ map

Table S1. Comparison of the the proportion of LCZs between the global LCZ map and that generated from locally derived TAs

|  | Global LCZ map | | Locally generated LCZ | |
| --- | --- | --- | --- | --- |
| LCZ | Pixel Count | Area cover(%) | Pixel Count | Area cover(%) |
| 1 | 0 | 0.0 | 209 | 0.1 |
| 2 | 83 | 0.0 | 237 | 0.1 |
| 3 | 51311 | 15.5 | 43317 | 16.7 |
| 4 | 8 | 0.0 | 0 | 0.0 |
| 5 | 95 | 0.0 | 2638 | 1.0 |
| 6 | 107287 | 32.5 | 111222 | 42.8 |
| 7 | 593 | 0.2 | 33498 | 12.9 |
| 8 | 12004 | 3.6 | 3395 | 1.3 |
| 9 | 158391 | 48.0 | 64363 | 24.8 |
| 10 | 477 | 0.1 | 737 | 0.3 |


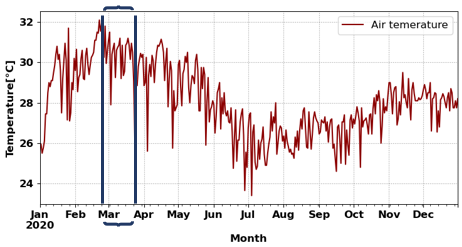


Fig. S2: Annual time series of air temperature for year 2020 at Lagos Airport

**2. Anthropogenic Heat**

To have a more realistic representation of the anthropogenic heat (Q_F_) generated in Lagos, we ran a simple standalone Surface Urban energy and water balance Scheme(SUEWS) using Python (SuPy: Grimmond, 2020; Sun & Grimmond, 2019). The model was driven with the urban canopy information of the local climate zone scheme and forced with ERA5 surface meteorology at 500m spatial resolution and population data from Grid^3^ (https://grid3.gov.ng/). SUEWS calculates Q_F_ based on heating and cooling degree days and population density (Järvi et al., 2011). The resulting values of anthropogenic heat per grid cell were aggregated by each LCZ and used in the UCMs model (Table S1).

Table S2. Urban canopy, Radiative and thermal urban parameters per LCZ class, compiled from Stewart et al., (2012), (Stewart et al.,(2014), and Wouters et al., (2016).

|  |  | | | **Albedo α(-)** | | | **Emissivity**$\in$(-)  [Unit Fraction] | | | **Vol heat capacity***10^6^  **(J m^-3^ K^-1^)** | | | **Heat conductivity**  **(J m^-1^ s^-1^ K^-1^)** | | |
| --- | --- | --- | --- | --- | --- | --- | --- | --- | --- | --- | --- | --- | --- | --- | --- |
|  | ISA  [Unit Fraction] | BH | SUEWS- Q_F_ | Roof | Walls | Road | Roof | Walls | Road | Roof | Walls | Road | Roof | Walls | Road |
| LCZ1 | 0.95 | 37.50 | 20.5 | 0.13 | 0.25 | 0.14 | 0.91 | 0.90 | 0.95 | 1.80 | 1.80 | 1.75 | 1.25 | 1.09 | 0.77 |
| LCZ2 | 0.90 | 17.50 | 71.1 | 0.18 | 0.20 | 0.14 | 0.91 | 0.90 | 0.95 | 1.80 | 2.67 | 1.68 | 1.25 | 1.50 | 73.00 |
| LCZ3 | 0.85 | 6.50 | 53.1 | 0.15 | 0.20 | 0.14 | 0.91 | 0.90 | 0.95 | 1.44 | 2.05 | 1.63 | 1.00 | 1.25 | 0.69 |
| LCZ5 | 0.70 | 17.50 | 13.7 | 0.13 | 0.25 | 0.14 | 0.91 | 0.90 | 0.95 | 1.80 | 2.00 | 1.50 | 1.25 | 1.45 | 0.62 |
| LCZ6 | 0.60 | 6.50 | 20.7 | 0.13 | 0.25 | 0.14 | 0.91 | 0.90 | 0.95 | 1.44 | 2.05 | 1.47 | 1.00 | 1.25 | 0.60 |
| LCZ7 | 0.85 | 3.00 | 17.7 | 0.13 | 0.20 | 0.14 | 0.28 | 0.90 | 0.95 | 2.00 | 0.72 | 1.67 | 2.00 | 0.50 | 0.72 |
| LCZ8 | 0.85 | 6.50 | 17.0 | 0.18 | 0.25 | 0.14 | 0.91 | 0.90 | 0.92 | 1.80 | 1.80 | 1.38 | 1.25 | 1.25 | 0.51 |
| LCZ9 | 0.30 | 6.50 | 1.5 | 0.13 | 0.25 | 0.14 | 0.91 | 0.90 | 0.95 | 1.44 | 2.56 | 1.37 | 1.00 | 1.00 | 0.55 |
| LCZ10 | 0.55 | 10.00 | 4.9 | 0.10 | 0.20 | 0.14 | 0.91 | 0.90 | 0.95 | 2.00 | 1.69 | 1.49 | 2.00 | 1.33 | 0.61 |

Note: ISA: Impervious Surface Area, BH : Building Height, SUEWS-Q_F_ (Anthropogenic heat flux from SUEWS)

**3.0 Urbanization Impact on Urban heat pattern**

To distinguish the impacts of urbanization and sea breeze on air temperature patterns, we conducted a no-urban case simulation. In this simulation, we replaced the urban pixels with vegetation while keeping the same time period. Analyzing the daytime Urban Heat Island (UHI) pattern, as shown in Fig. S4(c), we observed that the temperature in the core urban areas was higher than in the simulation with urban presence. This finding underscores the significant influence of urban morphology on the air temperature pattern. The presence of tall buildings structures in urban areas contributes to reduced radiation trapping through reflections, thereby affecting the temperature distribution. Furthermore, we noticed that areas closer to the coast experienced colder temperatures than those located further inland. This cooling effect could be attributed to the advection of cool sea breeze from the coast, impacting the local temperature distribution.


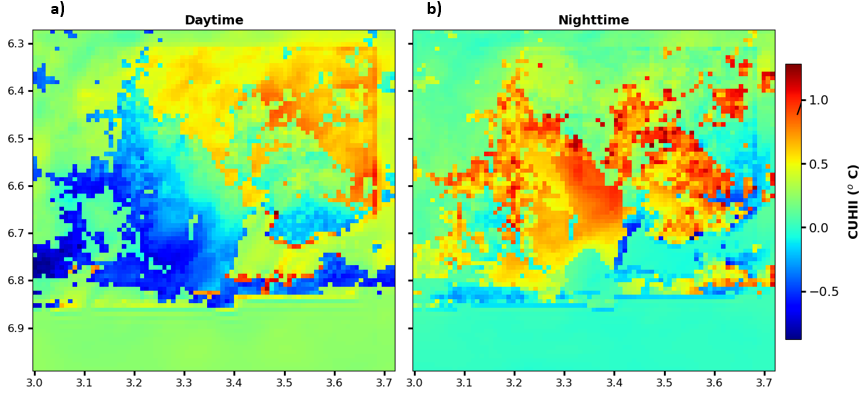


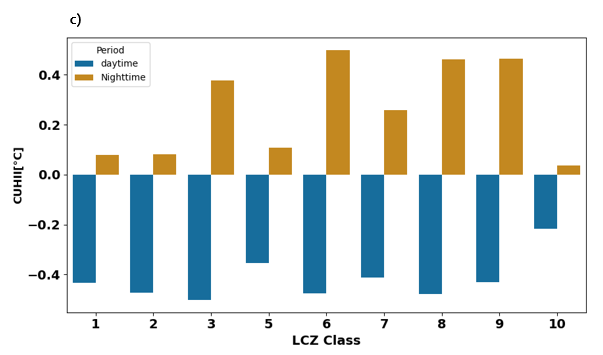


Fig. S4 Spatial pattern of urban Heat Island during the a) day, b) Night and c) a grouped bar plot comparing the day and nighttime UHI in each urbanized LCZs

**4. Heat stress Analysis**

**4.1 A comparison of heat Indices: Humidex vs WBGT**

The Wet-Bulb Globe Temperature, WBGT (Yaglou & Minard, 1957) on the other hand is widely considered as the most frequently used heat stress index due to its straightforward physical interpretation and its derivation from a broader range of environmental factors such as air temperature, humidity, wind, and solar radiation.

WBGT is formulated as a linear combination of the natural wet-bulb temperature, $T_{w}$, black globe temperature$, T_{g}$ and air temperature,$T_{2}$:

$WBGT = {0.7T}_{w}+ {0.2T}_{g}+ {0.1T}_{2 (1)}$

Where $T_{w}$ is estimated from relative humidity and air temperature using Stull’s equation (Stull, 2011) and $T_{g}$ measures the combined effect of radiant heat $L_{w}$, air temperature $T_{2}$, and wind speed $ws$.

$T_{g}$= $T_{2}{+ 0.017L}_{w}-0.0208ws (2)$

Obviously, WBGT will seem to be a better representation of human comfort than Humidex since it would account for the effect of four environmental factors than Humidex’s two. However, recent studies (e.g Budd, 2008; Havenith & Fiala, 2016; Kong & Huber, 2022) have shown that WBGT responds poorly to humidity and wind speed and consequently underestimates the heat stress under restricted evaporation in a prevailing highly humid environment. In this regard, it is possible that the humidity and wind patterns might not be well accounted for in a complex coastal city like Lagos using WBGT. Nevertheless, we compared the 2 indices and found a good linear relation between them as shown in Fig. S5 (R^2^ = ~0.7).


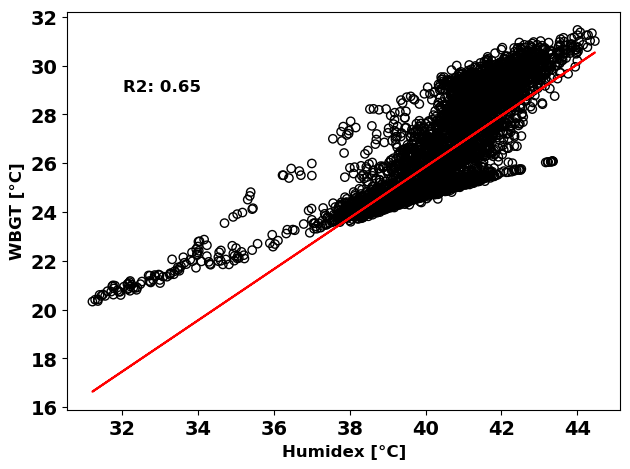


Fig.S5- Scatter plot showing the linear relation between WBGT and Humidex (^O^C)

**5. Model evaluations**

**5.1 Air Temperature Evaluation**


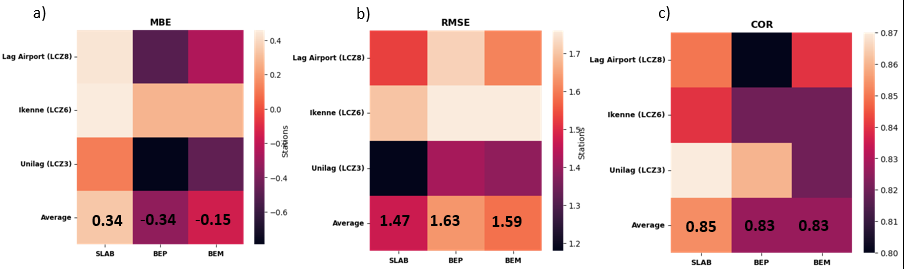


Fig.S6- Heat maps showing a) the MBE B) RMSE and c) the correlation coefficients of predicted T_2_ (^o^C) and observations over the selected stations.

**5.2 Wind Speed**

The wind pattern is shown in Fig. S7 corresponding to Ikenne (LCZ 6), Lagos Airport (LCZ8) and Unilag (LCZ 3). Overestimation of the diurnal pattern of wind speed is observed in Ikenne by all the models. The overestimation is most pronounced in the SLAB model, followed by BEP and least in BEP+BEM model. In Lagos Airport, we noted a general underestimation of wind speed, particularly during the day. Additionally, we noted that BEP model was able to capture the low wind speed at nighttime than SLAB and BEP+BEM model. In Unilag location, the diurnal pattern of wind speed by BEP model seems to be closest to observations, whereas the overestimations of the diurnal wind pattern is observed in the SLAB model and underestimations by the BEP+BEM. Despite the similarities in the physical configurations of BEP and BEP+BEM model, we found a general low wind speed in diurnal pattern of wind speed predicted by BEP+BEM . This might be related to the addition of a drag coefficient in the latest version of WRF(V>4.3: Zonato & Chen, 2021). The drag coefficient influences momentum sinks caused by vertically oriented surfaces (buildings and vegetation) as well as turbulent kinetic energy generated by buildings (Martilli et al., 2002). The drag from horizontal surfaces is however based on the MOST. In previous versions, drag coefficients were constant, however in WRFV4.3, this was modified to depend on the building plan area ratio, since the ratio affects the overall drag in simulations using Reynolds-averaged Navier-Stokes equations (Santiago & Martilli, 2010 ; Gutiérrez et al., 2015). The low wind speed predictions by BEP+BEM has also been noted by (Hendricks & Knievel, 2022) over Houston.


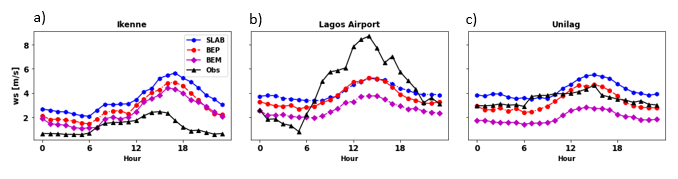


Fig. S7- Comparison of mean diurnal pattern of 10m air at the 3 locations

The error metrics evaluation between observed and modelled wind speed for each location and aggregates indicated a general overestimation in all the models with largest MBE occurring in the SLAB model (MBE=1.63), this is followed by BEP model (MBE=0.97m/s). However, BEP+BEM predicted wind speed closer to 0m/s (MBE= 0.05m/s). Likewise, BEP+BEM presents the lowest RMSE (RMSE =2.19m/s), followed by BEP (RMSE = 2.6m/s) and the highest error is noted with the SLAB model (RMSE = 3.1m/s). The correlation between the observed and predicted wind speeds is generally low with a correlation coefficient of 0.32, 0.36 and 0.35 for SLAB, BEP and BEP+BEM models respectively. The heat map for the evaluation metrics for the three stations is shown in Fig. S8. Although with a grid resolution of 1 km, we might expect the WRF model to perform better in simulating wind speed, as noted by Solbakken et al., (2021). However, the low correlation values observed between the model output and observations may be related to the model's land-use specification at each location. For instance, at Lagos Airport, the model grid with a resolution of 1 km is classified as LCZ 8, which represents large low-rise buildings. In reality, the meteorological site is located on grass with the closest low-rise buildings at least 100m away.


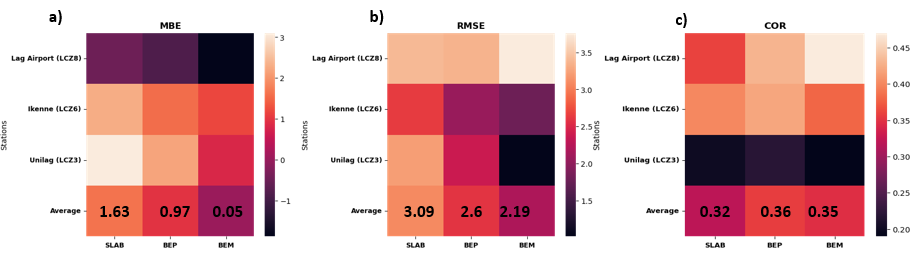


Fig. S8- Heat maps showing a) the MBE B) RMSE and c) the correlation coefficients of predicted wind speeds and observations over selected stations.

**5.3 Wind Direction and Wind speed frequency**

Fig. S9-S11 illustrates the comparison of modelled and observed wind directions and wind speed frequency. Over Ikenne (LCZ6) location. The prevalent wind direction is southwesterlies, which the models did well in replicating. However, BEP+BEM model shows much more variability in the wind direction than SLAB and BEP model. The wind speed histogram shows a general overestimation of the wind speed. Low wind speed frequency were underestimated (0-1m/s) by all the models, 1% by SLAB, 4% by BEP however BEP+BEM shows an improved performance with 14%. Wind speeds between 1 -2mls were well captured by BEP and BEP+BEM. 35% of the observed wind speed are between 1-2m/s, BEP predicted 34%, and BEP+BEM predicted 33% whereas the SLAB model predicted just 17**%.** It is evident that the model successfully captured the dominant sectors in the direction, indicating that the direction sectors that were not accurately simulated by the model have little significance.


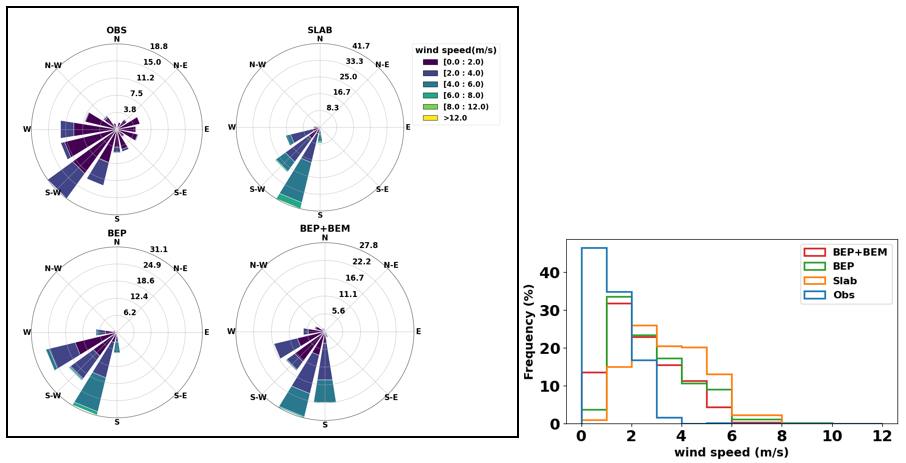


Fig. S9a) wind rose and b) wind speed histogram depicting the wind speed frequencies in Ikenne location

Over Lagos airport, because this is an airport weather station, wind speed less than 3m/s which is classified as 0(calm). For comparison, modelled wind speed less than 3m/s were also classified as calm. The prevalent wind direction from observation is southwesterlies, which the models did well in replicating. However, BEP+BEM model shows much more variability in the wind direction than SLAB and BEP model. The wind speed histogram shows a general overestimation of the wind speed. Low wind speeds frequency were underestimated (0-1m/s) by all the models, 1% by SLAB, 4% by BEP however BEP+BEM shows an improved performance with 14%. Wind speeds between 1 -2mls were well captured by BEP and BEP+BEM. 35% of the observed wind speed are between 1-2m/s, BEP predicted 34%, and BEP+BEM predicted 33% whereas the SLAB model predicted just 17**%.** It is evident that the model successfully captured the dominant sectors in the direction, indicating that the direction sectors that were not accurately simulated by the model have little significance.


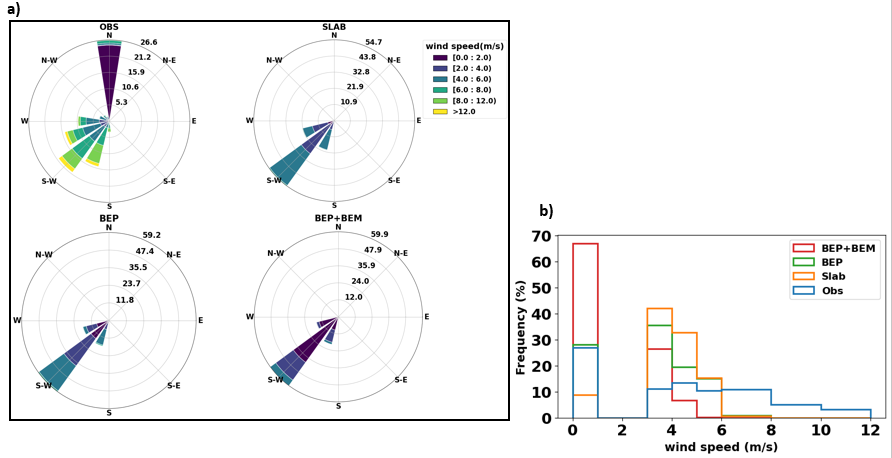


Fig. S10 a) Wind rose and b) wind speed histogram depicting the wind speed frequencies in Lagos airport location.

However, in Unilag location where north-westerly winds predominate, the 3 models failed to reproduce the wind directions as they all predicted southwesterlies. This again might be due to mismatch between the actual description of the measurement site and the grid description. Moreover, BEP+BEM did well in capturing the wind speed frequencies than BEP and SLAB. We noted that majority of the wind speed are between 1-2m/s (90%). BEP+BEM predicted the highest percentage within this range. As low wind speeds were observed in the location for (90%). BEP+BEM predicted the highest percentage within this range with a frequency of 50% followed by BEP (5%) and SLAB with 1%.


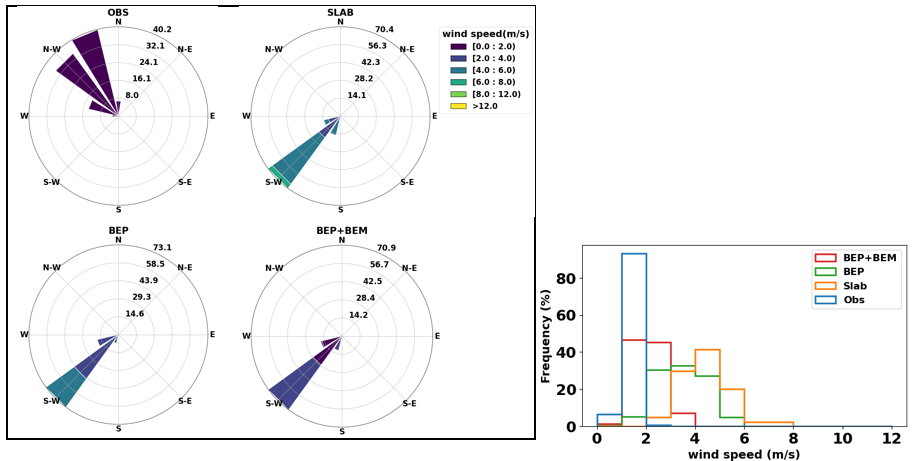


Fig. S11- a) wind rose and b) wind speed histogram depicting the wind speed frequencies over Unilag location


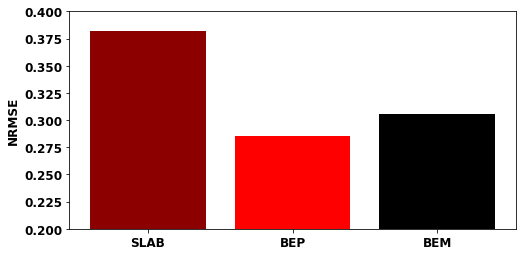


*Fig. S12 Bar chart illustrating the normalised root mean square error for air temperature, wind speed and relative humidity.*

.
